# Association Between Rheumatoid Arthritis and Risk of Parkinson's Disease: A Meta-Analysis and Systematic Review

**DOI:** 10.3389/fneur.2022.885179

**Published:** 2022-05-11

**Authors:** Dongxiu Li, Xia Hong, Tingyu Chen

**Affiliations:** Department of Nursing, Fujian Health College, Fuzhou, China

**Keywords:** rheumatoid arthritis, Parkinson's disease, risk, epidemiology, meta-analysis, systematic review

## Abstract

**Background:**

Rheumatoid arthritis (RA) and Parkinson's disease (PD) are two common chronic diseases worldwide, and any potential link between the two would significantly impact public health practice. Considering the current inconsistent evidence, we conducted a meta-analysis and systematic review to examine the risk of PD in patients with RA.

**Methods:**

Two investigators (DL and XH) conducted a comprehensive search of PubMed, Embase, and Web of Science using medical subject headings terms combined with free words to identify relevant papers published from inception through December 31, 2021. All studies that explored the relationship between RA and PD were included for quantitative analysis and qualitative review. Random- and fixed-effects models were used to pool the risk ratios (RRs) of PD in patients with RA. The Newcastle-Ottawa scale was used to assess the quality of included studies. This study followed the Preferred Reporting Items for Systematic Reviews and Meta-Analyses (PRISMA 2020) guideline.

**Results:**

Four population-based studies involving 353,246 patients and one Mendelian randomized study were included in our study. The pooled result showed a significantly reduced risk of PD in patients with RA than in the general population (RR = 0.74, 95% CI: 0.56-0.98, *P* = 0.034). No apparent effects of gender, age, region, follow-up time, or study design on PD risk were observed. Sensitivity analysis showed that pooled results were relatively stable, and no publication bias was detected. The Mendelian randomization study indicated a significant inverse association between RA and PD (genetic correlation: −0.10, *P* = 0.0033) and that each one standard deviation increase in the risk of RA was significantly associated with a lower risk of PD. Of note, the current study is limited by the relatively small number of included studies and unmeasured confounding factors, especially for RA-related anti-inflammatory agents.

**Conclusions:**

This study supports that people with RA had a lower PD risk than those without RA. Further studies are needed to explore the underlying molecular mechanisms of the interaction between the two diseases.

## Introduction

Parkinson's disease (PD) is a chronic progressive motor dysfunction characterized by static tremor, rigidity, and bradykinesia (1–4). As the second most common neurodegenerative disease globally, PD affects 1-2 of every 1,000 people; its prevalence increases with age, with up to 1% of people over the age of 60 suffering from PD (5). PD is associated with severe loss of dopamine neurons in the substantia nigra pars compacta and depletion of dopamine in the basal ganglia leading to motor deficits, and the accumulation of misfolded α-synuclein in the inclusion bodies forming Lewy bodies is a typical neuropathological manifestation of PD (6, 7). The specific mechanisms underlying the pathogenesis of PD are unknown and may be the result of environmental, lifestyle, and genetic interactions (8, 9). Although PD is a brain disease, the initial pathogenic events may occur in the gut and peripheral olfactory system (10–12). There is growing evidence that inflammation and immunity play an important role in the pathogenesis of PD (13, 14).

Rheumatoid arthritis (RA) is an inflammatory autoimmune disease. Similar to PD, RA is a common chronic disease that affects approximately 1% of people worldwide (15). RA can cause joint stiffness, swelling, pain, and decreased mobility and also affect organs outside the joints, such as the skin, eyes, lungs, and even the central nervous system (16). The presence of hyperactivation of proinflammatory mediators and immune disorders in RA patients theoretically contributes to the pathogenesis of PD (17). In addition, previous studies suggested a possible overlap in the genetic risk of PD and autoimmune diseases (18). Several epidemiological studies have explored the risk of PD in patients with RA, but the results are inconsistent (19–21).

Both RA and PD are highly prevalent diseases worldwide, and any correlation between the two would have important implications for public health practice and potentially provide new targets and ideas for the management of PD. Considering the current conflicting evidence and the lack of a study comprehensively summarizing current data, we conducted this meta-analysis and systematic review to elucidate the impact of RA on PD risk and provide higher quality evidence.

## Materials and Methods

This study was reported following the Preferred Reporting Items for Systematic Reviews and Meta-Analyses (PRISMA 2020) guidelines (22). The protocol for this study was not registered.

### Search Strategies

Two investigators (DL and XH) independently searched PubMed, Embase, and Web of Science databases for relevant literature published from inception through December 31, 2021. The search strategy consisted of subject terms (e.g., rheumatoid arthritis, Parkinson's disease) and synonyms (e.g., arthritis deformans, chronic articular rheumatism, idiopathic parkinsonism, paralysis agitans) for RA and PD; the medical subject headings terms were linked by “AND” and the synonyms by “OR.” No language restrictions or filters were used. The specific search strategy for each database is available in the Supplementary Material. In addition, we manually browsed the reference lists of eligible papers and related reviews to identify additional eligible studies.

### Inclusion and Exclusion Criteria

Studies that fulfilled all of the following criteria would be considered for inclusion in meta-analysis: (1) RA and PD were clearly defined or met diagnostic criteria, such as the readable codes or standard clinical diagnostic criteria; (2) correlations between RA and risk of PD were explored; (3) studies reported relative risk estimates and corresponding 95% confidence intervals (CIs), such as risk ratio (RR), hazard ratio (HR), odds ratio (OR), and standardized incidence ratio (SIR), or provided sufficient data to calculate these ratios; and (4) the study design was a longitudinal observational study, including case-control and cohort studies, whether prospective or retrospective.

Studies meeting any of the following criteria would be excluded: (1) studies that did not generate original data, such as reviews, meta-analyses, commentaries; (2) non-human studies, such as cellular or animal model experiments; (3) case reports or case series of fewer than 10 patients; (4) overlapping study participants; for multiple studies from the same population, the study with the largest sample size and the most complete outcomes would be included.

Studies that did not fully meet the above criteria but explored the association between RA and risk of PD would be qualitatively analyzed as part of the systematic review.

### Data Extraction and Quality Assessment

Two investigators (DL and XH) independently screened the titles and abstracts of the initially retrieved records according to the eligibility criteria. Potentially eligible papers were read in full, and reasons for exclusion would be recorded.

For included studies, two researchers independently (DL and TC) extracted the following information: first author, publication year, study period, age, gender, population source, sample size, study design, effect size, methods of PD and RA identification, adjusted/matched confounders, and follow-up time.

The Newcastle-Ottawa scale (NOS) was used to assess the quality of included studies, and three main aspects were involved: (1) selection of participants in each group; (2) comparability between study groups; and (3) exposure determination in case-control studies and assessment of outcomes of interest in cohort studies (23). A study with a NOS score > 6 was considered to have low-risk bias; otherwise, it was considered a low-quality study.

### Statistical Analysis

We used fixed- and random-effects models to calculate pooled risk estimates of PD in RA patients compared to the non-RA population (24). For studies that reported both crude and adjusted effect sizes, the adjusted effect size was extracted for combining. Heterogeneity was judged using Cochran's *Q*-test and Higgins' *I*^2^ statistics; pooled results of the fixed-effects model were reported when *I*^2^ <50% and *P* > 0.1, otherwise, heterogeneity between the included studies was considered significant and results of the random-effects model were reported. We performed sensitivity analyses by excluding one study at a time and comparing the results of the fixed- and random-effects models. Publication bias was assessed by observing the symmetry of the funnel plot and performing Egger's and Begg's tests. We performed subgroup analyses according to age, sex, follow-up, region, and study design to explore the effects of these variables on the pooled results. Data for this study were analyzed using STATA/MP 16.0 (StataCorp LLC, TX, USA). All *P*-values were two-tailed, and the statistical significance threshold was 0.05.

## Results

### Study Selection

**Figure 1:**
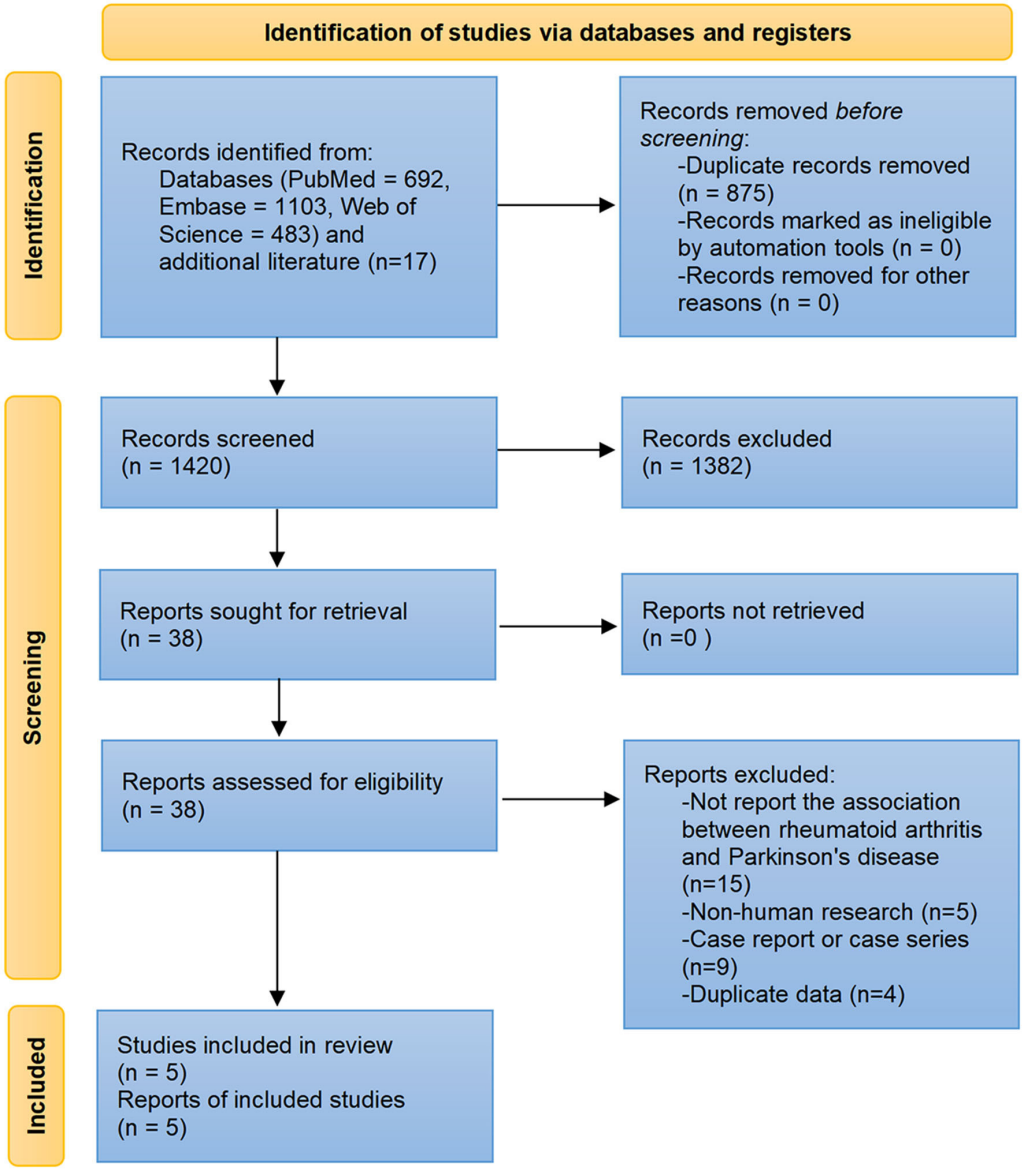
Flow diagram of the study selection process.

The predeveloped database search strategies and browsing the reference lists of relevant papers yielded 2,295 records. After removing 875 duplicates and excluding 1,382 apparently irrelevant papers, 38 papers were read in full. Among them, 15 did not report results of interest, 9 were case reports or case series, 5 were cellular or animal model studies, and 4 had duplicate data. A total of five studies were finally included in this review (Figure 1).

### Description of the Included Studies

Of the eligible studies, four were population-based studies, and one was the Mendelian randomization study (19–21, 25, 26). The four population-based studies involved 353,246 participants, of which two were from Sweden, one from Denmark, and one from China (19–21, 26). The Mendelian randomization study evaluated the genetic association between RA and PD using summary statistics from genome-wide association studies on RA of European ancestry (25). All population-based studies used International Classification of Disease (ICD) codes to identify PD and RA cases. The median/mean age was over 50 years, and the longest follow-up was up to 44 years. The detailed characteristics of the included studies are presented in Table 1.

**Table 1:** Characteristics of included studies.

**References**	**Region**	**Gender (male/** **female)**	**Mean/** **median age-yr**.	**Study design**	**Population**	**RA cohort/** **PD cases**	**Non-RA cohort/** **comparators**	**Period**	**Identification of RA**	**Identification of PD**	**Matching/** **adjustment confounder**	**Mean/** **median follow-up, yr**.
Sung et al. (19)	Taiwan, China	37,195/128,910	RA cohort: 53.9, non-RA cohort: 53.4	Cohort study	Taiwan NHIRD	33,221	132,884	1998-2011	ICD	ICD	Matched for age, sex, and year of RA diagnosis; adjusted for age, sex, NSAID use, and comorbidities (diabetes, hypertension, hyperlipidemia, coronary artery disease, head injury, depression, stroke).	RA cohort: 6.61, non-RA cohort: 6.70
Rugbjerg et al. (20)	Denmark	44,524/37,616	PD: 73.0; non-PD: 65-75	Case-control study	Danish National Hospital Register	13,695	68,445	1986-2006	ICD	ICD	Adjusted for chronic obstructive pulmonary disease	NP
Bacelis et al. (26)	Sweden	NP	PD cohort: median > 70; non-PD cohort: NP	Nested case-control study	Socialstyrelsen (the Swedish governmental agency managing medical registries)	4,738	47,269	1997-2016	ICD	ICD	Matched for birth year, sex, birth location, and time of follow-up	1-20
Li et al. (21)	Sweden	NP	NP	Cohort study	MigMed database (the center for primary health care research at the Lund University)	52,994	Standardized incidence ratios (Expected incidence rate)	1964-2007	ICD	ICD	Adjusted for age, period, socioeconomic status, region of residence, hospitalization of chronic obstructive pulmonary disease, and alcoholism and alcohol-related liver disease.	1-44
Li et al. (25)	European	NA	NP	Mendelian randomization study	Rheumatoid arthritis of European ancestry	Case (RA): 19,377; Case (PD): 33,647	Control (non-RA): 53,911; Control (non-PD): 449,056	NA	NA	NA	Removed single nucleotide polymorphisms searched in the GWAS Catalog that were reported to be associated with smoking and body mass index	NA

*RA, rheumatoid arthritis; PD, Parkinson's disease; NHIRD, National Health Insurance Research Database; NSAID, nonsteroidal anti-inflammatory drugs; ICD, International Classification of Diseases; GWAS, genome-wide association study; NA, not applicable; NP, not reported.*

The NOS scores of the included studies ranged from 7 to 9, indicating a high level of overall quality. These studies had clear exposure and outcome definitions, appropriate adjustment for confounders, and sufficiently long follow-up (Table 2).

**Table 2:** The quality assessment of included studies.

**Study (cohort)**	**Representativeness of exposed cohort**	**Selection of non-exposed cohort**	**Ascertainment of exposure**	**Outcome not present before study**	**Comparability**	**Assessment of outcome**	Follow-up long enough*	**Adequacy of follow up**	**Quality** **score**
Sung et al. (19)	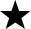	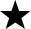	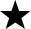	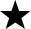	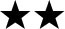	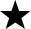	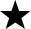	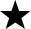	9
Li et al. (21)	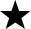	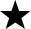	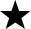	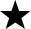	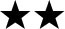	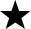	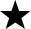	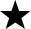	9
**Study (case-control)**	**Case definition**	**Representativeness of the cases**	**Selection of controls**	**Definition of controls**	**Comparability**	**Ascertainment of exposure**	**Same method of ascertainment**	**Non-response rate**	**Quality** **score**
Rugbjerg et al. (20)	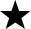	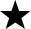	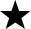	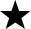	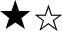	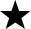	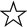	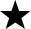	7
Bacelis et al. (26)	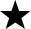	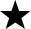	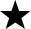	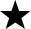	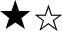	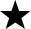	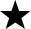	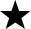	8

* *Median follow-up of more than 5 year or maximum follow-up of more than 10 years was assigned a star*.

### Overall Association Between RA and Risk of PD

**Figure 2:**
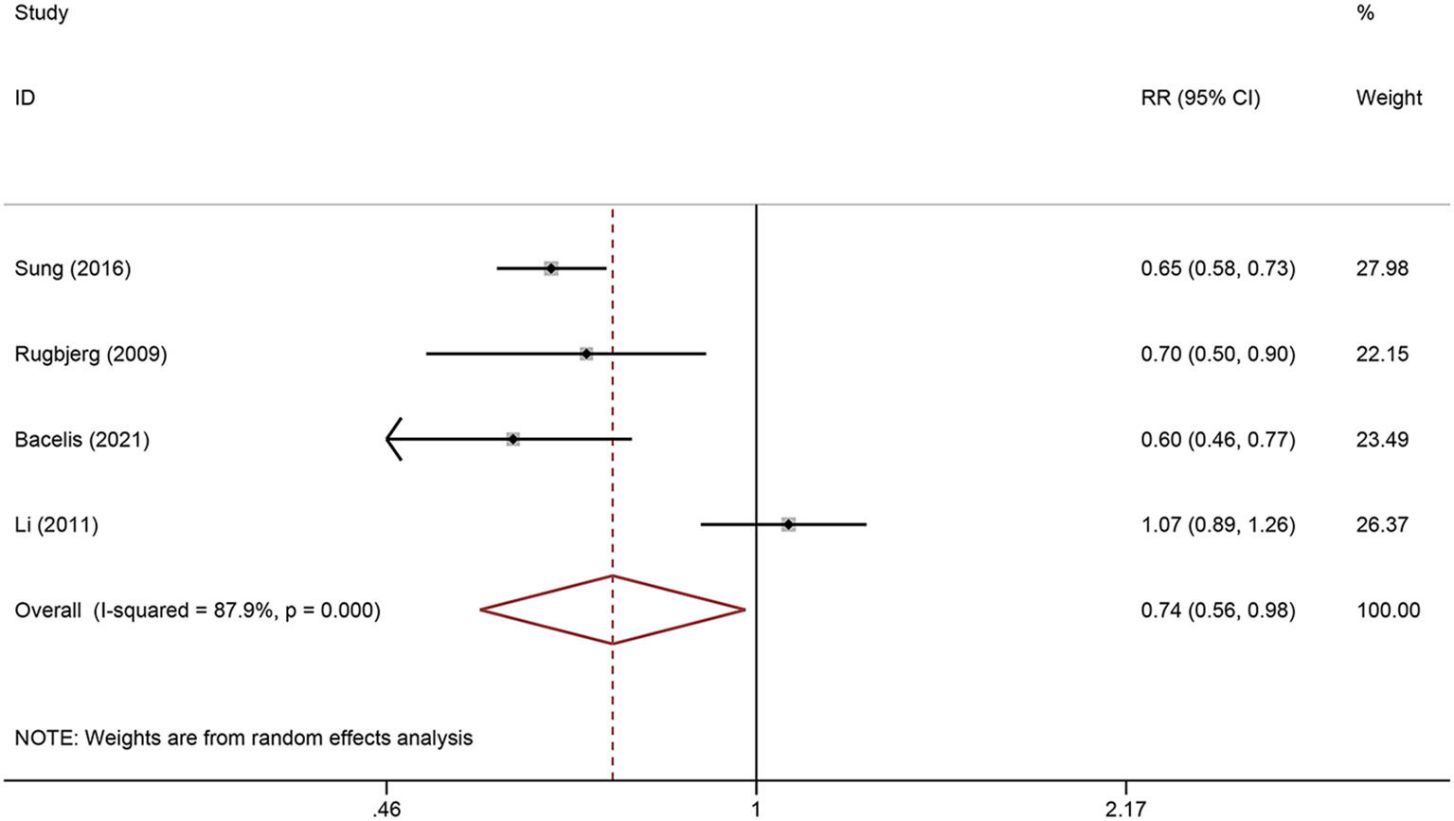
Forest plot of the associations between Rheumatoid arthritis and risk of Parkinson's disease.

All population-based studies were included in the meta-analysis. The heterogeneity test showed significant heterogeneity between included studies (I^2^ = 87.9%, P < 0.001), so the random-effects model was used (Figure 2). The pooled result from more than 300,000 participants showed a significantly lower risk of PD in RA patients than in the general population (RR = 0.74, 95% CI: 0.56-0.98, P = 0.034) (Figure 2).

The Mendelian randomization study conducted by Li et al. found a significant inverse genetic correlation between RA and PD (genetic correlation: −0.10, P = 0.0033). Mendelian randomization analysis was performed in two samples and showed that each one standard deviation increase in the risk of RA was significantly associated with a lower risk of PD (OR = 0.904, P < 0.001 and OR = 0.929, P < 0.001) (25).

### Subgroup Analysis

We performed stratified analyses according to sex, age (<65 vs. ≥65 years old), duration of follow-up (<5 vs. ≥5 years), region, and study design.

No significant differences in effect sizes were observed between the above subgroups, and RA was associated with a reduced risk of PD in all conditions. However, the pooled results for age subgroups (<65 years old: RR = 0.79, 95% CI: 0.50-1.25, P = 0.313; ≥65 years old: RR = 0.78, 95% CI: 0.58-1.04, P = 0.086), European region (RR = 0.77, 95% CI: 0.53-1.14, P = 0.191), and cohort studies (RR = 0.83, 95% CI: 0.51-1.35, P = 0.455) were not statistically significant; this may be due to the small number and substantial heterogeneity of the included studies resulting in conservative confidence intervals (Table 3).

**Table 3:** Stratified analysis of the association between rheumatoid arthritis and Parkinson's disease.

**Subgroup**	**Studies (*n*)**	**RR**	**95%CI**	***P*_overalleffect_**	**Heterogeneity (*I^**2**^*, *P_***Q***_*)**
**Gender**					
Male	3	0.64	0.53-0.78	<0.001	0.0%, 0.544
Female	3	0.66	0.58-0.74	<0.001	7.1%, 0.341
**Age**					
<65 years old	2	0.79	0.50-1.25	0.313	71.6%, 0.030
≥65 years old	2	0.78	0.58-1.04	0.086	72.0%, 0.013
**Followed-up**					
<5 years	1	0.65	0.56-0.76	<0.001	NA
≥5 years	4	0.68	0.61-0.77	<0.001	27.1%, 0.231
**Region**					
Asia	1	0.65	0.58-0.73	<0.001	NA
Europe	3	0.77	0.53-1.14	0.191	87.0%, <0.001
**Study design**					
Cohort study	2	0.83	0.51-1.35	0.455	95.4%, <0.001
Case-control study	2	0.64	0.53-0.78	<0.001	0.0%, 0.439

*RR, risk ratio; NA, not applicable*.

### Sensitivity Analysis

**Figure 3:**
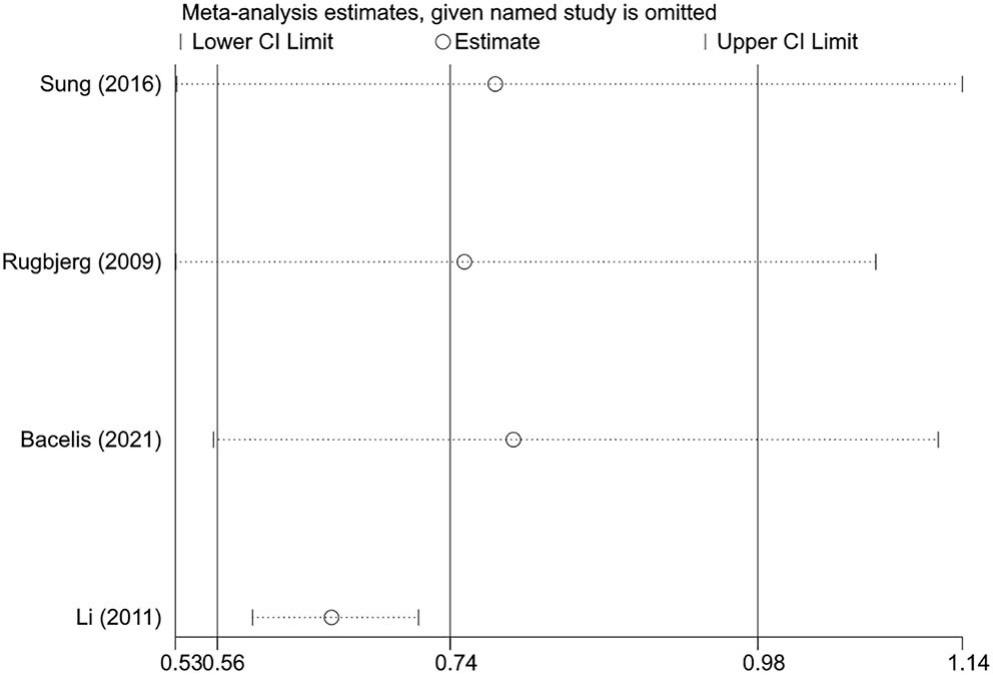
The effects of the individual studies on the pooled effect size of Parkinson's disease risk.

We performed sensitivity analyses to test the robustness of the combined results by two methods. First, the impact of individual studies on the overall pooled result was assessed by excluding one study at a time. Although the statistical significance of the pooled result disappeared after excluding certain studies, patients with RA still tended to have a lower risk of PD (Figure 3). In addition, we compared the results of random-effects and fixed-effects models for each meta-analysis. Interestingly, all results were statistically significant for the inverse correlation between RA and PD risk when using the fixed-effects model. The 95% CIs for the random-effects models passed through 1 in some subgroups, but the effect sizes were not markedly different from that of the fixed-effects model (Table 4). Overall, the association between RA and reduced risk of PD was relatively stable.

**Table 4:** Comparison of the results of random-effects vs. fixed-effects models.

**Analysis groups**	**RR (95% CI), fixed-effects model**	**RR (95% CI), random-effects model**
Total	0.73 (0.67-0.80)	0.74 (0.56-0.98)
Male	0.64 (0.53-0.78)	0.64 (0.53-0.78)
Female	0.66 (0.58-0.74)	0.66 (0.58-0.74)
<65 years old	0.70 (0.58-0.83)	0.79 (0.50-1.25)
≥65 years old	0.69 (0.61-0.78)	0.78 (0.58-1.04)
Followed-up <5 years	0.65 (0.56-0.76)	0.65 (0.56-0.76)
Followed-up ≥ 5 years	0.68 (0.61-0.77)	0.68 (0.59-0.78)
Asia	0.65 (0.58-0.73)	0.65 (0.58-0.73)
Europe	0.85 (0.75-0.97)	0.77 (0.53-1.14)
Cohort study	0.76 (0.69-0.83)	0.83 (0.51-1.35)
Case-control study	0.64 (0.53-0.78)	0.64 (0.53-0.78)

### Evaluation of Publication Bias

The funnel plot was not performed because of the small number of included studies at the end. The *P*-values for both Begg's and Egger's tests were >0.05 (0.734 and 0.881, respectively), indicating a low likelihood of potential publication bias.

## Discussion

To our knowledge, this is the first study to systematically assess the association between RA and PD risk. The pooled result from four population-based studies showed a 26% reduction in the risk of PD among patients with RA compared to the general population. Despite the small number of included studies, the results from high-quality studies and more than 300,000 participants can be considered a stringent estimate. A Mendelian randomization study provided further strong evidence for the inverse association between RA and risk of PD. Overall, the inverse association between RA and PD risk is relatively stable in populations with different characteristics.

There was significant heterogeneity in the current pooled results, which may be attributed to differences in study region, sample source, and study design. Of note, the heterogeneity of the overall meta-analysis disappeared when excluding the study by Li et al. (*I*^2^ = 0%, *P* = 0.737), and the pooled results remained statistically significant (RR = 0.65, 95% CI: 0.59-0.72, *P* < 0.001). The cohort study by Li et al. used a database covering the Swedish Hospital Discharge Register from 1964 to 2007, and standardized incidence ratios were calculated by the ratio of observed to expected number of cases; they found no correlation between RA and risk of PD (SIR = 1.07, 95% CI: 0.89-1.26) (21). Interestingly, another nested case-control study from Sweden, conducted by Bacelis et al. in a more recent period but not the same database, found that patients with RA had a significantly lower risk of PD (26). In the study by Li et al., PD cases were identified using hospital registries, and PD was the main diagnosis for hospitalization. However, PD patients with comorbid RA may be more susceptible to hospitalization, thus leading to detection bias. In addition, although the diagnosis of PD in the study by Li et al. was accurate, only patients with severe PD, not all PD cases, were hospitalized. Therefore, Li et al. may have grossly underestimated the risk of PD, and a more appropriate interpretation of their findings may be that there was no difference in the incidence of severe PD in patients with RA compared to the general population (21).

RA often requires treatment with anti-inflammatory drugs that may affect the correlation between RA and risk of PD. Drugs commonly used in RA patients are non-steroidal anti-inflammatory drugs (NSAIDs) and immunosuppressive drugs including traditional disease-modifying anti-rheumatic drugs (DMARDs) (such as methotrexate, hydroxychloroquine, cyclosporine, azathioprine, and leflunomide) and biologic DMARDs (such as adalimumab, rituximab, etanercept, abatacept, golimumab, and tocilizumab) (27, 28). Some epidemiological studies have suggested that NSAIDs may be protective against the development of PD (29, 30), and basic experiments also showed that NSAIDs could reduce the formation of neurotoxic molecules and inhibit dopaminergic neurotoxicity, thereby exerting neuroprotective effects (31–33). In addition, a previous study found that the use of tumor necrosis factor (TNF)-α inhibitors in patients with inflammatory bowel disease reduced the risk of PD by approximately 80% (34). Of the studies on the association between RA and PD, only Sung et al. explored the effects of NSAIDs and DMARDs; they found that NSAIDs were a protective factor for PD, but after controlling for NSAIDs use, RA patients still had a lower risk of developing PD compared to individuals without RA (HR = 0.65, 95%CI: 0.58-0.73). Stratified analysis suggested that the reduced risk of PD was also independent of DMARDs (with DMARDs use: HR = 0.64, 95% CI: 0.55-0.74 vs. without DMARDs: HR = 0.66, 95% CI: 0.57-0.77), while biologic DMARDs administration further reduced the risk of PD in RA patients (HR = 0.57, 95%CI: 0.41-0.79) (19). In patients with severe RA, sometimes steroids combined with DMARDs are prescribed to rapidly relieve the patient's joint symptoms and systemic inflammation; unfortunately, no studies explored the role of steroids in the link between RA and PD, and there is a lack of research on the effect of steroids on the risk of PD under other conditions. Furthermore, in some areas, herbal plants and phytoconstituents may be used to treat RA, some of which might affect the pathophysiological processes of PD, such as ursolic acid, chlorogenic acid, mucuna pruriens, tripterygium wilfordii glycosides, sinomenine (35–43). However, Mendelian randomization analysis using genetic instrumental variants did not support that reduced risk of PD in RA patients was attributed to treatment factors for RA (25). Thus, the reduced risk of PD in patients with RA cannot be fully explained by anti-inflammatory therapy, but it may deepen the association.

Although our meta-analysis and Mendelian randomization study demonstrated that RA patients have a lower risk of PD, the exact mechanism of the reduced risk is unclear. In fact, the current results contradict the hypothesis on the pathological mechanisms of RA and PD. Inflammation is thought to play a critical role in RA and PD, and long-term chronic inflammation and excessive production of inflammatory mediators such as interleukin (IL)-1, IL-6, TNF-α, and chemokines may trigger inflammatory processes and activate microglia in the brain, leading to degeneration of dopaminergic neurons and an increased risk of PD (13, 16, 44–46). Combined with the above discussion, anti-inflammatory drugs did not fully explain the reduced risk of PD in RA patients, suggesting the association between RA and PD through mechanisms other than inflammation. The manifestation of inflammation in the brain of PD patients may be primarily related to the progression of the disease or its compensated consequence rather than affecting its onset. Therefore, there are several other possible explanations for our results as follows.

First, the association between RA and PD may be influenced by lifestyle factors. A classic example is smoking; smoking is an important risk factor for RA but is associated with a reduced risk of PD (9, 47, 48). The current studies were adjusted using only chronic obstructive pulmonary disease as a surrogate variable for smoking and did not directly include smoking data of individuals. Other unmeasured confounding factors, including coffee intake and level of education, also influence the risk of both diseases (48–50). In addition, RA mostly develops in middle age, whereas PD tends to occur after the age of 60 (9, 51). Therefore, behavioral changes after RA diagnosis, such as reduced pesticide exposure, controlled alcohol intake, and increased attention to healthcare, may impact subsequent PD risk. Second, RA and PD may have similar clinical presentations; the presentation of RA masks symptoms of PD, or rheumatologists are not sufficiently aware of PD symptoms, so PD may be underdiagnosed in RA patients. Third, there may be genetic antagonistic pleiotropy that exhibits dual effect phenotypes. That is, increased risk of RA and decreased risk of PD (or vice versa) (25). Moreover, it has been recently shown that lysosomal dysfunction may lead to α-synuclein aggregation and the formation of Lewy bodies, a pathological feature of PD, while lysosomal activity is increased in RA (25, 52–54). Therefore, there may be a protective effect on PD pathogenesis through the lysosomal pathway in RA patients, and the exploration of its targets may provide new ideas for PD prevention and treatment.

The strength of our study is that the pooled results from large sample size studies could provide a stringent estimate. Although there may be multiple theoretical mechanisms for the reduced risk of PD, the implications of anti-inflammatory drugs associated with RA are of interest. Due to limited data, subgroup analyses stratified by medications were not performed, but the study by Sung et al. provided evidence that biologic DMARDs use may reduce the risk of PD (19). Previous studies have shown that proinflammatory cytokines such as IL-1, IL-6, and TNF-α, which are targets of biologic agents for RA, are elevated in patients with PD and may be related to its pathogenesis (55–57). Therefore, elucidating the underlying molecular mechanisms of interaction between peripheral and central immunity may help provide new targets for the treatment of PD. Moreover, choosing safe anti-inflammatory drugs and lifestyle with anti-inflammatory effects may have a protective effect on PD risk.

There are several limitations to this study that should be noted. The main limitation is the relatively small number of included studies, but the large sample and high-quality characteristics of these studies partially compensate for this and make the conclusions relatively stable. All included studies were derived from medical record databases and used readable codes to identify cases, whereas diagnostic criteria for PD and IBS may be inconsistent and subject to misdiagnosis and underdiagnosis. In addition, as discussed above, the results of observational studies may be biased by unmeasured and unconsidered variables.

## Conclusion

According to the meta-analysis of population-based studies and the Mendelian randomization study, patients with RA had a lower risk of developing PD than those without RA. The current results may be partially explained by anti-inflammatory drugs related to RA. Further studies are needed to explore the underlying molecular mechanisms of the interaction between the two diseases, which may help provide new insights into prevention and therapeutic targets for PD.

## Data Availability Statement

The original contributions presented in the study are included in the article/Supplementary Materials, further inquiries can be directed to the corresponding author/s.

## Author Contributions

DL contributed to the conception and design of the study. DL and XH performed the database search and study selection. DL and TC extracted data, conducted data analysis, and wrote the manuscript. XH checked and verified all data. All authors contributed to manuscript revision, read, and approved the submitted version.

## Conflict of Interest

The authors declare that the research was conducted in the absence of any commercial or financial relationships that could be construed as a potential conflict of interest.

## Publisher's Note

All claims expressed in this article are solely those of the authors and do not necessarily represent those of their affiliated organizations, or those of the publisher, the editors and the reviewers. Any product that may be evaluated in this article, or claim that may be made by its manufacturer, is not guaranteed or endorsed by the publisher.
