# Supplementary material for: Association Between Rheumatoid Arthritis and Risk of Parkinson's Disease: A Meta-Analysis and Systematic Review
Source: Front Neurol. 2022 May 11;13:885179. doi: 10.3389/fneur.2022.885179 (PMC9130734; doi:10.3389/fneur.2022.885179)
Supplement: Supplementary file 1 [file Table_1.DOCX]

| **The exact search strategy for each database** | |
| --- | --- |
| **PubMed** | ("Arthritis, Rheumatoid"[Mesh] or Rheumatoid Arthritis or arthritis deformans or arthritis, rheumatoid or arthrosis deformans or beauvais disease or chronic articular rheumatism or chronic polyarthritis or chronic progressive poly arthritis or chronic progressive polyarthritis or chronic rheumatoid arthritis or disease, beauvais or infantile rheumatoid arthritis or inflammatory arthritis or polyarthritis, primary chronic or primary chronic polyarthritis or rheumarthritis or rheumatic arthritis or rheumatic polyarthritis or rheumatism, chronic articular) AND ("Parkinson Disease"[Mesh] or Idiopathic Parkinson's Disease or Lewy Body Parkinson's Disease or Parkinson's Disease, Idiopathic or Parkinson's Disease, Lewy Body or Parkinson Disease, Idiopathic or Parkinson's Disease or Idiopathic Parkinson Disease or Lewy Body Parkinson Disease or Primary Parkinsonism or Parkinsonism, Primary or Paralysis Agitans) |
| **Embase** | ('rheumatoid arthritis'/exp or 'arthritis deformans':ab,ti OR 'arthritis, rheumatoid':ab,ti OR 'arthrosis deformans':ab,ti OR 'beauvais disease':ab,ti OR 'chronic articular rheumatism':ab,ti OR 'chronic polyarthritis':ab,ti OR 'chronic progressive poly arthritis':ab,ti OR 'chronic progressive polyarthritis':ab,ti OR 'chronic rheumatoid arthritis':ab,ti OR 'disease, beauvais':ab,ti OR 'infantile rheumatoid arthritis':ab,ti OR 'inflammatory arthritis':ab,ti OR 'polyarthritis, primary chronic':ab,ti OR 'primary chronic polyarthritis':ab,ti OR 'rheumarthritis':ab,ti OR 'rheumatic arthritis':ab,ti OR 'rheumatic polyarthritis':ab,ti OR 'rheumatism, chronic articular') AND ('parkinson disease'/exp OR 'idiopathic parkinsonism':ab,ti OR 'lewy bodies of parkinson disease':ab,ti OR 'lewy bodies of parkinsons disease':ab,ti OR 'lewy body parkinson disease':ab,ti OR 'lewy body parkinsons disease':ab,ti OR 'paralysis agitans':ab,ti OR 'parkinson dementia complex':ab,ti OR 'parkinsons disease':ab,ti OR 'primary parkinsonism':ab,ti) |
| **Web of Science** | TS=((Arthritis, Rheumatoid or Rheumatoid Arthritis or arthritis deformans or arthritis, rheumatoid or arthrosis deformans or beauvais disease or chronic articular rheumatism or chronic polyarthritis or chronic progressive poly arthritis or chronic progressive polyarthritis or chronic rheumatoid arthritis or disease, beauvais or infantile rheumatoid arthritis or inflammatory arthritis or polyarthritis, primary chronic or primary chronic polyarthritis or rheumarthritis or rheumatic arthritis or rheumatic polyarthritis or rheumatism, chronic articular) AND  (idiopathic parkinsonism or Lewy bodies of Parkinson disease or Lewy bodies of Parkinson's disease or Lewy bodies of Parkinsons disease or Lewy body Parkinson disease or Lewy body Parkinson's disease or Lewy body Parkinsons disease or paralysis agitans or Parkinson dementia complex or Parkinson's disease or Parkinsons disease or primary parkinsonism or Parkinson disease)) |
